# Acute Care Surgery: Navigating Recent Developments, Protocols, and Challenges in the Comprehensive Management of Surgical Emergencies

**DOI:** 10.7759/cureus.52269

**Published:** 2024-01-14

**Authors:** Kiran Prasad Moparthi, Herra Javed, Monika Kumari, Peddi Pavani, Antonella Paladini, Ayesha Saleem, Raja Ram, Giustino Varrassi

**Affiliations:** 1 General Practice, California Institute of Behavioral Neurosciences and Psychology, Fairfield, USA; 2 Graduate, Shifa College of Medicine, Islamabad, PAK; 3 Surgery, Liaquat National Hospital and Medical College, Karachi, PAK; 4 General Surgery, Kurnool Medical College, Andhra Pradesh, IND; 5 Department of MESVA, University of L'Aquila, L'Aquila, ITA; 6 General Surgery, Hayatabad Medical Complex (HMC), Peshawar , PAK; 7 Medicine, MedStar Washington Hospital Center, Washington, USA; 8 Pain Medicine, Paolo Procacci Foundation, Rome, ITA

**Keywords:** acute care surgery and trauma, surgical retreatment, surgical emergencies, acs, acute care

## Abstract

Acute care surgery (ACS) is a crucial medical field that specifically deals with the rapid treatment of surgical emergencies. This investigation encompasses the most recent progress, procedures, and obstacles in ACS, utilizing various sources such as scholarly articles, clinical trials, and expert statements. The development of ACS as a specialized field is a significant area of concentration, particularly emphasizing its contribution to improving patient care. An examination is conducted on the efficacy of contemporary triage systems and prompt response mechanisms, specifically in diminishing the incidence of illness and death rates associated with illnesses such as trauma, acute appendicitis, and obstructed viscera. The emphasis is placed on the surgical protocols and principles that form the basis of ACS. Examining regional and international approaches provides insight into the distinctions and commonalities in surgical techniques. An assessment is conducted to determine the effects of the transition to minimally invasive procedures on patient outcomes, recuperation periods, and healthcare expenses. The assessment also examines the logistical obstacles that ACS encounters, such as resource allocation and managing diverse teams. The examination focuses on the delicate equilibrium between prompt decision-making and care grounded in evidence. It also evaluates the possible contribution of technical breakthroughs such as telemedicine and AI to improving patient care and overcoming current obstacles. The topic of training and education for surgeons in ACS is of utmost importance and requires careful consideration. The evaluation evaluates the sufficiency of existing educational frameworks and the necessity of specific training to equip surgeons for the requirements of ACS. This analysis explores the current discourse surrounding the standardization of ACS training, considering its potential ramifications for the future of surgical procedures. Exploring ethical and legal problems in ACS also includes situations when prompt decision-making may clash with patient autonomy and informed consent. The significance of proficient communication with patients and their families during emergency surgical scenarios is underscored, emphasizing the necessity for ethical awareness and interpersonal aptitude. The investigation of ACS demonstrates its dynamic character, signifying notable advancements while recognizing enduring obstacles. Continual research, interdisciplinary collaboration, and policy adjustments are necessary to improve ACS procedures. This thorough investigation offers valuable insights for professionals and researchers, facilitating future progress in managing surgical crises.

## Introduction and background

Acute care surgery (ACS) is a specialized surgical area that prioritizes the prompt evaluation and management of urgent and emergent surgical problems. It comprises many therapeutic techniques that treat life-threatening surgical requirements, such as trauma surgery, critical care, and emergency surgery. ACS is distinguished by its promptness; timely intervention is crucial for patient outcomes [1]. This field encompasses the administration of injuries and conditions such as traumatic brain injuries, abdominal emergencies, acute appendicitis, and obstructed viscera, among other examples. ACS includes providing postoperative care to patients and handling complications that may arise after emergency surgical interventions [2]. The scope of ACS extends beyond the surgical process itself. The process encompasses preoperative assessment, intraoperative judgment, and postoperative management. In addition, ACS practitioners frequently collaborate with other medical disciplines to deliver complete care to patients in emergency settings [3]. Implementing this interdisciplinary strategy is essential to guaranteeing the most favorable results in intricate and time-critical circumstances.

The beginnings of ACS can be attributed to the early methodologies employed in trauma surgery and critical care. Traditionally, the management of patients requiring immediate surgical attention was divided and frequently distributed among many surgical specialties [4]. The development of ACS was significantly influenced by trauma surgery. The establishment of structured trauma systems during the mid-20th century, particularly in the period surrounding and following World War II, represented a notable change in the approach to providing urgent surgical care. Military surgeons pioneered methodologies and guidelines to address injuries incurred during armed conflicts, transferring numerous of these approaches to civilian medical procedures [5]. The advancement of emergency surgical care was significantly accelerated in the 1960s and 1970s with the introduction of trauma centers and the use of improved life support procedures in the civilian sector [6]. The American College of Surgeons' Committee on Trauma (ACS-COT) played a crucial role in establishing trauma care and education benchmarks by creating the Advanced Trauma Life Support (ATLS) program.

The necessity for a specialized field dedicated to emergency surgery unrelated to trauma became more apparent during the late 20th and early 21st centuries. As a result, ACS was formalized as a distinct specialty. In 2005, the American Association for the Surgery of Trauma introduced a new model for trauma and emergency surgical treatment, which resulted in the formation of ACS as an acknowledged subspecialty [7]. Notable breakthroughs in surgical methodologies and technologies also characterize the progress of ACS. Minimally invasive techniques, including laparoscopy and endoscopy, have become increasingly common in ACS, providing advantages such as shorter recovery periods and decreased rates of complications [8]. The merger of modern imaging technology with interventional radiology has also altered the management of acute surgical problems, allowing for more accurate diagnosis and less intrusive treatment alternatives.

The development of ACS has been dramatically influenced by training and education. Specialized fellowship programs have been established to equip surgeons with the requisite expertise and understanding to operate in this challenging domain [9]. These programs prioritize not only surgical methods but also critical care, trauma treatment, and the organization of multidisciplinary care teams. The development of ACS exemplifies a broader transition in the medical domain towards enhanced and tailored treatment for intricate and pressing medical disorders. ACS continues to lead in managing surgical emergencies, maintaining a position of innovation and excellence as healthcare systems progress [10]. The progression of ACS is propelled by persistent research, technical improvements, and an unshakable dedication to enhancing patient outcomes. The sector is evidence of the ever-changing nature of medicine and the unwavering commitment to improving healthcare for individuals with pressing surgical requirements [9].

## Review

Methodology

This methodology presents a detailed strategy for performing a narrative literature review using the Scale for the Assessment of Narrative Review Articles (SANRA) parameters. These guidelines are crucial for guaranteeing a methodical and exhaustive study. The approach commences by identifying a distinct and defined research question that serves as a guiding principle for the extent and orientation of the review. The importance of this question lies in its ability to ensure that the literature search and subsequent analysis are customized to fulfill specific objectives and are in line with current gaps or controversies in the field. The literature search approach is a crucial component of this process. The process entails establishing criteria for inclusion and exclusion, which are determined by characteristics such as the publication date, language, publishing type, and relevance to the research issue. A complete search utilizes keywords and subject headings in selected databases and resources such as PubMed, Scopus, and Web of Science. The search technique is thoroughly recorded, including the specific terms utilized and the number of articles obtained. This rigorous documentation ensures transparency and reproducibility.

After doing the literature search, selecting sources entails a methodical screening process. This procedure involves a first evaluation that relies on the titles and abstracts of publications to eliminate irrelevant ones. It is followed by a thorough examination of possibly pertinent articles in their entirety to ascertain their appropriateness for inclusion. An established mechanism is in place to address any conflicts among reviewers during this selection process. The essence of this methodology lies in the extraction and synthesis of data. Each article undergoes a structured analysis to extract crucial information, including study aims, techniques, primary findings, and conclusions. The synthesis entails conducting a thematic analysis, which entails finding and discussing recurring patterns, topics, and significant insights pertinent to the research issue. Integrating findings from individual studies into a coherent knowledge of the topic depends on this stage, which is of utmost importance.

The included articles undergo a quality check to ensure the trustworthiness and accuracy of the review conclusions. This evaluation considers variables such as the thoroughness of the research methods, the clarity of the study's presentation, and the applicability of the findings. The narrative synthesis subsequently combines these discoveries, examining key motifs, study inconsistencies, and ramifications for the discipline. Additionally, it identifies deficiencies in the existing body of literature and proposes potential avenues for future research. The presentation and reporting of the review adhere to the SANRA checklist, guaranteeing transparency and clarity in reporting. The review comprises an abstract succinctly presenting the main findings and implications, a meticulous methodology section, a total synthesis of the existing literature, a discussion that interprets the findings from a broader perspective, and a conclusion that summarizes the essential ideas. Including a rigorous peer-review procedure is essential to this methodology since it guarantees the high standard and precision of the review. The feedback provided by peer reviewers is utilized to further develop and enhance the review. Due to the ever-changing nature of scientific research, the review is regularly updated to include discoveries and ensure its continued relevance.

Recent developments in acute care surgery

The field of ACS has had significant breakthroughs in recent years, propelled by technological innovations, novel surgical techniques, and progress in pharmacology. These advancements have had a substantial influence on the management of surgical crises, resulting in enhanced patient outcomes and more streamlined care provision [1]. Implementing technological developments has played a pivotal role in the transformation of ACS. Incorporating digital technologies in emergency surgical care has fundamentally transformed how surgeons approach, diagnose, and manage acute surgical diseases. The utilization of high-definition imaging and diagnostic instruments is a notable technological achievement [2]. Advanced imaging modalities, including computed tomography (CT) scanning, magnetic resonance imaging (MRI), and ultrasound, have undergone significant advancements, resulting in improved resolution and enhanced visualization of inside injuries and medical disorders. The enhanced imaging capabilities have empowered surgeons to make precise diagnoses, crucial in time-sensitive emergency scenarios [3].

Telemedicine has been adopted as a significant technological advancement in ACS. Telemedicine enables remote consultations and diagnostics, which is especially advantageous in rural or underserved regions with limited access to ACS professionals [4]. Telemedicine enables surgeons to provide remote guidance to local medical teams in stabilizing patients and conducting essential interventions before transferring them to specialist centers. This enhances the prompt medical attention provided to patients and facilitates the strategic anticipation and arrangement for their admission to advanced medical facilities, optimizing the whole treatment procedure. Robotics and automated systems are now being utilized in ACS [5]. Robotic surgical systems, such as the da Vinci Surgical System, have gained popularity and are now utilized more frequently in various surgical procedures. Although their use in emergency surgery is still developing, these technologies have the potential to provide enhanced accuracy and manipulation, particularly in intricate procedures [6]. Moreover, using automated technologies for activities such as drug dispensation and patient surveillance has improved the effectiveness and precision of healthcare in acute surgical environments [6].

The advancements in surgical procedures have had a similarly profound impact on ACS. An essential domain of progress lies in the extensive implementation of minimally invasive surgical (MIS) procedures. MIS, which encompasses techniques like laparoscopy and endoscopy, has gained widespread popularity owing to its advantages, including shorter recovery periods, decreased susceptibility to infections, and smaller surgical incisions [7]. Utilizing MIS procedures can significantly enhance patient outcomes in emergency surgeries, where swift recuperation is paramount. For example, laparoscopic appendectomy and cholecystectomy have become established practices in numerous hospitals, providing patients with a faster recuperation period and reduced postoperative discomfort in comparison to conventional open surgeries [8]. Advancements in reconstructive and regenerative procedures have also been observed in the field of ACS. Recent advancements in wound closure and tissue restoration techniques have significantly enhanced the healing process for patients undergoing trauma and emergency surgery. Novel biomaterials and tissue engineering techniques are being created to facilitate the restoration of injured tissues, mitigating the lasting consequences of surgical procedures [9]. Not only have there been advancements in technology and surgery, but there has also been notable progress in pharmacology regarding ACS. New medicine formulations and delivery systems have improved the control of pain and infection, which are crucial components of postoperative care [10]. An example is the utilization of extended-release local anesthetics and multimodal pain management techniques, which have improved pain management after surgery. This has resulted in faster patient recovery and decreased dependence on opioids.

The emphasis on antibiotic stewardship has significantly increased in ACS, particularly in light of the growing prevalence of antibiotic-resistant illnesses. Effective utilization of novel antibiotic regimens and the prudent application of current antibiotics have played a pivotal role in managing infections in postoperative patients. Furthermore, ongoing research is focused on advancing targeted antimicrobial medicines tailored to particular patient profiles [11]. This holds the potential for more efficient and individualized infection control. The progress in pharmacology extends beyond pain management and infection control. Advancements in hemostatic medicines have significantly improved the management of traumatic bleeding, a frequent obstacle encountered in emergency surgical procedures [12]. Hemostatic medications and other treatments that promote coagulation have proven essential in quickly controlling bleeding, leading to improved survival rates in cases of trauma and acute care surgery. Moreover, the significance of pharmacogenomics in ACS is increasingly being recognized as a crucial field of study [13]. Comprehending the impact of genetic variants on individual drug reactions can result in the development of more tailored and efficient medication plans for surgical patients. This individualized strategy not only enhances the effectiveness of treatment but also reduces the likelihood of negative medication responses, a notable concern in the demanding setting of ACS [14]. The field of ACS is undergoing tremendous changes because of technological developments, innovations in surgical procedures, and progress in pharmacology. These advancements alter how acute surgical emergencies are handled, improving patient outcomes and providing more streamlined care. Continued research and innovation will be crucial in tackling remaining problems and enhancing patient care in critical surgical scenarios as the area progresses [15].

Current protocols in surgical emergencies

Standard operating procedures (SOPs) play a crucial role in the dynamic and vital field of ACS. These SOPs guarantee that patients experiencing surgical emergencies receive prompt, streamlined, and successful treatment [16]. This discussion explores the different facets of SOPs in the field of ACS, encompassing triage and prioritization strategies, protocols for managing patients during surgery, interdisciplinary approaches, collaboration with other medical specialties, the involvement of critical care, and the significance of ancillary services such as radiology and laboratory testing [17].

Triage and Prioritization Strategies

Triage and prioritizing are the fundamental principles of effectively addressing surgical emergencies. The main goal is to identify the patients with the highest level of illness severity and guarantee they receive prompt medical attention. Triage protocols are commonly directed by frameworks such as the ATLS standards, which classify patients according to the gravity of their injuries and the immediacy of their requirement for surgical treatment [18]. These tactics aim to optimize resource utilization and minimize treatment time, vital for enhancing patient outcomes. Efficient triage necessitates a prompt yet comprehensive evaluation of a patient's state, frequently in difficult and time-sensitive situations [19]. This procedure involves assessing the patient's vital signs, the severity of their injuries, and the probability of survival with prompt action. Subsequently, prioritization procedures are utilized to ascertain the sequence in which patients are attended to, considering the gravity of their diseases and the accessible resources [20].

Intraoperative Management Protocols

The management of patients with ACS during surgery is a multifaceted and crucial aspect of patient care. Intraoperative care protocols are formulated to maximize patient outcomes while ensuring the safety and efficiency of surgical procedures [21]. These guidelines include various areas, such as surgical procedures, anesthesia administration, blood and fluid revival, and the supervision and handling of probable complications. Intraoperative protocols encompass decision-making processes about the necessary scope of surgery. Damage control surgery (DCS) may be the recommended method in certain situations, particularly for patients who are seriously ill [22]. This entails conducting the essential operations to stabilize the patient, followed by subsequent definitive surgery once the patient's condition has been stabilized.

Interdisciplinary approach in acute care surgery

ACS necessitates a meticulously synchronized interdisciplinary approach encompassing diverse healthcare professions and disciplines. This cooperative approach guarantees that every area of patient care is considered, ranging from the initial evaluation and stabilization to the surgical procedure and subsequent postoperative care [22]. The interdisciplinary team comprises surgeons, anesthesiologists, emergency medicine physicians, nurses, and other healthcare specialists. Every team member plays a vital part in providing patient care, utilizing their specialized knowledge to oversee the patient's treatment effectively [23]. Efficient communication and coordination among team members are essential to guarantee smooth and thorough patient care.

Collaboration with Other Specialties

Cooperation with different medical disciplines is essential for efficiently handling surgical crises. This entails collaborating closely with experts in disciplines such as radiology, neurosurgery, orthopedics, and critical care. These partnerships are crucial for delivering all-encompassing healthcare to patients with intricate and diverse injuries or diseases [24]. Radiologists are essential in the initial evaluation and continuous monitoring of patients, as they provide critical imaging data that informs surgical decision-making [24]. Neurosurgeons and orthopedic surgeons have a role in treating traumatic brain injuries and complex fractures, respectively, by offering specialist surgical care as part of the comprehensive therapy strategy.

Significance of Critical Care

The significance of critical care in ACS cannot be exaggerated. Patients experiencing surgical emergencies frequently necessitate thorough observation and assistance in an acute care environment, either before or after the operation. Urgent care entails meticulously managing the patient's essential bodily processes, such as providing respiration and circulatory activity aids, addressing infections, and vigilantly monitoring for any problems [25]. Critical care also continuously evaluates the patient's state, which helps inform subsequent treatment choices. The urgent care team collaborates closely with the surgical team to ensure seamless treatment and enhance patient outcomes [25].

Importance of Ancillary Services

ACS relies on ancillary services, such as radiography and laboratory tests, as crucial elements. Radiology services offer essential diagnostic information that informs surgical planning and action. Advanced imaging modalities, such as CT scans and MRI, provide intricate visualizations of inside damage, facilitating precise diagnosis and strategic treatment planning [26]. Laboratory services are of similar importance since they provide vital information about the patient's physiological condition. This encompasses hematological analyses to evaluate hemorrhaging, organ functionality, and infection, among other variables. Prompt and precise laboratory findings are crucial for making well-informed decisions regarding patient care, such as determining the necessity of blood transfusions, antibiotic treatment, and other interventions [27]. Overall, the SOPs in ACS are thorough and diverse, covering triage and priority, management during surgery, collaboration across different disciplines, and incorporating critical care and ancillary services. These standards are crucial for guaranteeing that patients with surgical emergencies receive the utmost treatment, enhancing their likelihood of recuperation and survival [28].

Challenges in acute care surgery

Acute care surgery is a discipline known for its high stakes, quick decision-making, and the requirement for accuracy in stressful situations. Although the field has undergone significant changes, it still encounters several obstacles that affect the quality of patient care, the allocation of resources, and the overall effectiveness of surgical procedures. The issues encompassed in this context involve the management of intricate cases, the resolution of ethical dilemmas, the handling of resource allocation and constraints, and the assurance of patient safety and quality assurance [28]. A significant obstacle in the field of ACS is the effective management of intricate surgical cases, frequently including patients with numerous injuries or pre-existing medical issues. These instances necessitate considerable proficiency and synchronization among multiple surgical and medical teams. The requirement for expeditious decision-making and intervention additionally heightens the intricacy [29]. Surgeons must promptly evaluate the patient's state, prioritize injuries, and determine the most efficient course of action, frequently with insufficient information. This situation requires expertise in surgery, a deep comprehension of multiple medical fields, and the capacity to make crucial judgments while facing intense stress [30].

Ethical Considerations in Emergency Surgery

Moral quandaries are a recurrent and consequential obstacle in ACS. During emergencies, patients frequently arrive in an unconscious state or in a condition where they are unable to give consent for surgical interventions. These circumstances give rise to inquiries regarding patient self-governance and the provision of informed consent [31]. Surgeons and medical teams must balance the imperative need for medical intervention with the imperative to uphold patient rights and preferences. This scenario becomes more intricate in instances involving end-of-life determinations or when the patient's desires are uncertain or ambiguously articulated [32].

Resource Allocation and Limitations

Resource allocation presents a notable obstacle in ACS. This encompasses tangible assets such as surgical suites, medical apparatus, and blood reserves and intangible assets such as skilled surgeons, nurses, and auxiliary personnel. Resource scarcity is prevalent in healthcare settings, especially in low- and middle-income countries or rural regions [33]. Surgeons frequently encounter the challenge of delivering optimal treatment under resource constraints, affecting the quality of care and patient outcomes. Furthermore, there is a need to reinforce the order of importance for patients when resources are limited, which requires making decisions based on medical and ethical considerations [34].

Patient safety and quality control

Prioritizing patient safety and upholding quality control are of utmost importance in ACS. This entails implementing policies and guidelines to mitigate errors and complications during surgical procedures. Nevertheless, keeping these principles can prove arduous in the fast-paced and unexpected setting of emergency surgery [34]. Emergency surgeries carry a greater risk of infection, surgical complications, and aftercare concerns when compared to elective treatments. Hence, it is imperative to create resilient systems to ensure quality control and safeguard patient safety [35].

Ensuring Patient Safety in Emergencies

To guarantee the safety of patients in emergency surgical scenarios, hospitals and surgical teams must enforce stringent safety measures. This encompasses strict adherence to sterilizing protocols, meticulous patient identification procedures, and the implementation of checklists as a preventive measure against errors [36]. In addition, ongoing training and simulations can equip surgical teams with the necessary skills to handle different emergencies, improving their capacity to deliver safe and efficient healthcare.

Quality Assurance in Surgical Practices

Surgical practices regularly monitor and evaluate surgical results, patient satisfaction, and procedural compliance as part of quality assurance. Additionally, it encompasses the execution of optimal methodologies and ongoing enhancement procedures [37]. Quality assurance in ACS is complex because of the wide range and intricate nature of instances. Nevertheless, it is vital to identify areas that require enhancement, optimize patient care, and mitigate difficulties.

Risk Management Strategies

Efficient risk management is crucial in ACS to minimize potential consequences and enhance patient outcomes. This entails the identification of potential hazards at different phases of surgical treatment, ranging from preoperative evaluation to postoperative management [37]. Risk management options encompass comprehensive patient evaluation, judicious implementation of prophylactic interventions such as antibiotics and venous thromboembolism prophylaxis, and vigilant surveillance for indications of problems. Ultimately, ACS encounters numerous complex issues that necessitate a comprehensive approach to tackle them effectively [38]. Effectively handling intricate cases, resolving moral quandaries, maximizing resource distribution, and guaranteeing patient safety and excellence are all essential elements of proficient acute care surgery. To address these problems and enhance outcomes for patients receiving emergency surgical care, engaging in continuous research, education, and policy creation is crucial as the field continues to advance [35].

Training and education in acute care surgery

Training and education are essential foundations in ACS, guaranteeing that practitioners possess the requisite knowledge, abilities, and experience to handle intricate and frequently life-threatening surgical crises [34]. The crucial element of ACS involves a wide range of educational components, including residency and fellowship programs, ongoing medical education, simulation and skill enhancement, and examining case studies and clinical results.

Residency and Fellowship Programs

Residency and fellowship programs are essential for surgical training, serving as the fundamental basis for surgeons developing their ACS skills. These programs are designed to provide extensive experience in various emergency surgical conditions, including trauma surgery, critical care, and elective surgical crises [39]. Residency programs primarily emphasize comprehensive surgical training, whereas fellowships provide more specific study in ACS. ACS fellowship programs are rigorous and often last for one to two years [40]. These programs aim to offer specialized training in handling surgical emergencies, encompassing advanced operating abilities, making important decisions under high-pressure circumstances, and managing patients in critical condition. These programs sometimes include rotations in other interconnected disciplines, such as trauma surgery, emergency medicine, and urgent care, to offer a comprehensive training experience [32].

Continuing Medical Education

Practicing surgeons must engage in continuing medical education (CME) to be updated on the newest advancements in ACS. Continual education is crucial to the swift progress of surgical methods, technologies, and patient care protocols. CME activities encompass a variety of formats, including conferences, workshops, seminars, and online courses [24]. These activities serve as a means to receive updates on the most recent research and achievements and create a venue for surgeons to engage in discussions regarding complex cases and exchange their experiences.

Simulation and Skill Development

The significance of simulation-based training in ACS education has been steadily growing. It offers a secure and hazard-free setting for surgeons to practice and enhance their expertise. Advanced simulations, such as virtual reality and patient simulators, provide accurate representations of situations that surgeons may face during surgical procedures [26]. These simulations are highly beneficial for honing intricate and uncommon procedures, augmenting decision-making abilities, and strengthening team communication and cooperation in emergencies [27].

Case studies and clinical outcomes

Examining case studies and evaluating clinical outcomes is essential to acquiring knowledge in ACS. Analyzing and debating actual cases aids in comprehending the intricacies of patient care, the process of making decisions, and the implementation of surgical methods in different scenarios [28]. This research also examines problems and bad outcomes, which are crucial for acquiring knowledge and enhancing future practice. Examining noteworthy instances in ACS offers valuable perspectives into the intricacies and difficulties of emergency surgical care [29]. These cases frequently function as standards for debating optimal methodologies, groundbreaking strategies, and crucial lessons. Notable instances also serve as a platform for evaluating the implementation of novel technologies and methods in a practical context [24].

Analysis of Success Stories and Complications

Examining both achievement and challenge is essential for comprehending the complete range of ACS. Success stories exemplify exceptional treatment and results, demonstrating efficacious management tactics and pioneering ideas. Conversely, it is equally crucial to analyze scenarios that involve complexity [30]. These evaluations aid in identifying possible obstacles, areas that can be enhanced, and measures to avoid similar problems in the future.

Outcome Metrics and Patient Feedback

Evaluating the quality of surgical care and education requires using outcome measurements and patient feedback, which are crucial tools. Outcome measures, such as morbidity and death rates, duration of hospitalization, and readmission rates, offer objective data regarding clinical performance [31]. Conversely, patient feedback provides valuable information on the patient's experience, level of happiness, and areas that need improvement, all from a patient-centric viewpoint. Ultimately, the training and education in ACS are complex and constantly developing. Residency and fellowship programs establish the foundation for surgical proficiency [32]. CME, simulation training, and case study studies enhance a surgeon's abilities and knowledge. An exhaustive examination of prominent cases, achievements, complexities, and patient input plays a crucial role in propelling the field of ACS forward, ultimately resulting in enhanced patient outcomes and elevated standards of care [34].

Future directions in acute care surgery

The field of ACS is continuously changing due to groundbreaking research, expanding methodologies, and the unwavering commitment to enhancing patient outcomes. As we contemplate the future, numerous trends and aspirations are influencing the course of this vital field [35].

Research Trends

Contemporary studies in ACS primarily concentrate on improving patient outcomes using technical breakthroughs and a more comprehensive comprehension of surgical pathophysiology. A significant development is the incorporation of precision medicine into surgical treatment [36]. This entails customizing surgical procedures according to specific patient attributes, such as genetic profiles, to enhance results and minimize the likelihood of problems. Ongoing research is focused on comprehending the biological reaction to surgical stress to devise approaches that reduce postoperative consequences, such as infections or organ failure [37]. Another emerging research area involves the utilization of big data and artificial intelligence (AI) in the field of surgery. Researchers are utilizing AI and machine learning to forecast patient outcomes, customize treatment plans, and improve decision-making during emergency scenarios, thanks to the growing accessibility of extensive healthcare datasets [37]. Artificial intelligence algorithms are currently being created to aid in diagnosing medical conditions, determining their associated risks, and even offering immediate guidance during surgical operations [38]. There is also an increasing interest in the sector for less-invasive procedures used in emergency surgery. Research investigates the practicability, security, and results of techniques such as laparoscopic and robotic surgery in urgent situations. The objective is to expand the advantages of these minimally invasive methods - such as diminished discomfort, accelerated healing, and decreased infection rates - to the high-risk setting of ACS [39].

Emerging Practices and Techniques

The development of new approaches in ACS is primarily motivated by enhancing patient care while effectively addressing the difficulties associated with emergency surgery. An example of such a technique is the implementation of Enhanced Recovery After Surgery (ERAS) procedures for emergency surgery patients [24]. The guidelines were initially designed for elective surgeries and aimed to optimize preoperative preparation, intraoperative management, and postoperative care to accelerate the healing process. Implementing these procedures for ACS could significantly improve patient outcomes and decrease hospitalization durations. An additional developing trend involves incorporating interdisciplinary teams in administering surgical emergencies [13]. These teams, comprising surgeons, anesthetists, nurses, and other professionals, collaborate to deliver complete treatment. This cooperative method efficiently handles patients with intricate ailments or many injuries. Regarding methods, there is a growing focus on DCS in ACS cases [25]. DCS, or damage control surgery, is a surgical approach that focuses on stabilizing the patient rather than doing a complete anatomical repair in cases of severe trauma. The utilization of a sequential surgical procedure has demonstrated enhanced survival rates in severely injured individuals. It is increasingly being adopted as a customary procedure in numerous trauma centers [40].

Predictions and Expectations for the Field

In the future, the field of ACS is anticipated to further progress towards providing treatment more tailored to individual needs and incorporating innovative technology. One forecast suggests that there will be greater integration of technological advancements into ACS, namely robots and artificial intelligence [22]. The integration is anticipated to augment surgical accuracy, enhance diagnostic precision, and offer superior predictive models for patient outcomes. Furthermore, it is anticipated that ACS training and education will change. Due to the increasing complexity of the sector, there are likely more specialized training programs that specifically target the intricacies of emergency surgical care. Simulation-based training and virtual reality are anticipated to impact these programs significantly, offering learners authentic and diverse experiences without any potential harm to patients [15]. ACS is expected to emphasize global health as a significant advancement. With the progress of the field, there is an increasing acknowledgment of the necessity to tackle inequalities in surgical treatment on a global scale. There is a high probability that there will be an increase in international cooperation, training initiatives, and the commitment of resources to enhance ACS capacities in low- and middle-income nations [19]. The future of ACS is characterized by promising advancements and an ongoing dedication to enhancing patient care. Research trends, new practices, and projections indicate that the profession is moving towards a more advanced and patient-focused discipline incorporating sophisticated technology [20]. As these events progress, ACS is ready to address the difficulties of surgical emergencies with enhanced effectiveness and inventiveness.

## Conclusions

In conclusion, ACS has experienced significant progress, propelled by technological changes, surgical methodologies, and pharmaceutical breakthroughs. Critical areas like minimally invasive treatments, telemedicine, and enhanced triage and prioritization tactics have transformed the sector, improving patient outcomes and efficiency in surgical emergencies. The future of ACS is anticipated to experience additional expansion as emerging trends, including precision medicine, artificial intelligence, and global health initiatives, hold the potential to transform surgical care. ACS remains at the vanguard of medical innovation, providing new possibilities and hope in the crucial care of surgical emergencies as it develops.
